# Glucose transporter GLUT1 expression is important for oriental river prawn (*Macrobrachium nipponense*) hemocyte adaptation to hypoxic conditions

**DOI:** 10.1016/j.jbc.2022.102748

**Published:** 2022-11-24

**Authors:** Xichao Sun, Cheng Xue, Yiting Jin, Chao Bian, Na Zhou, Shengming Sun

**Affiliations:** 1 Key Laboratory of Exploration and Utilization of Aquatic Genetic Resources, Ministry of Education, Shanghai Ocean University, Shanghai, China; 2 International Research Center for Marine Biosciences at Shanghai Ocean University, Ministry of Science and Technology, Shanghai, China; 3 Shenzhen Key Lab of Marine Genomics, Guangdong Provincial Key Lab of Molecular Breeding in Marine Economic Animals, BGI Academy of Marine Sciences, BGI Marine, BGI, Shenzhen, China; 4 State Key Laboratory of Quality Research in Chinese Medicine and School of Pharmacy, Macau University of Science and Technology, Macau, China

**Keywords:** GLUT1, invertebrate, hypoxia, glycolysis, glucose transport, cDNA, complementary DNA, ChIP, chromatin immunoprecipitation, DO, dissolved oxygen, GLUT, glucose transporter, HIF, hypoxia-inducible factor, HK, hexokinase, HRE, hypoxia-response element, IgG, immunoglobulin G, LDH, lactate dehydrogenase, OCR, oxygen consumption rate, PA, palmitic acid, PDK1, pyruvate dehydrogenase kinase isozyme 1, qRT–PCR, quantitative RT–PCR, ROS, reactive oxygen species, TCA, tricarboxylic acid

## Abstract

Crustaceans have an open vascular system in which hemocytes freely circulate in hemolymph. Hemocytes are rich in hemocyanin, a specific oxygen-transport protein in crustaceans; therefore, understanding the response of hemocytes to hypoxia is crucial. Although hemocytes take up glucose during hypoxia, the molecular mechanism of glucose uptake in crustaceans remains unclear. Herein, we identified two highly conserved glucose transporters (GLUT1 and GLUT2) in *Macrobrachium nipponense* (oriental river prawn) and analyzed their tissue-specific expression patterns. Our immunofluorescence assays showed that GLUT1 and GLUT2 are located on the cell membrane, with a strong GLUT1 signal in primary hemocytes under hypoxia. We found that during acute hypoxia, hypoxia-inducible factor-1α–related metabolic alterations result in decreased mitochondrial cytochrome *c* oxidase activity, implying a classic glycolytic mechanism. As a proof of concept, we replicated these findings in insect S2 cells. Acute hypoxia significantly induced hypoxia-inducible factor-1α, GLUT1, and pyruvate dehydrogenase kinase isozyme 1 expression in primary hemocytes, and hypoxia-induced increases in glucose uptake and lactate secretion were observed. *GLUT1* knockdown induced intracellular reactive oxygen species generation and apoptosis *in vitro* and *in vivo*, resulting in increased prawn mortality and more apoptotic cells in their brains, implying a vital function of GLUT1 in hypoxia adaptation. Taken together, our results suggest a close relationship between hypoxia-mediated glycolysis and GLUT1 in hemocytes. These results demonstrated that in crustaceans, adaptation to hypoxia involves glucose metabolic plasticity.

Oxygen has low solubility in water (only 3% of the equivalent volume of air), yet is indispensable for aquatic animals' behavior, development, and survival (1). Hypoxia occurs when dissolved oxygen (DO) falls below 2.8 mg/l in aquatic environments (2). During evolution, aquatic animals have acquired complex mechanisms to detect and respond to hypoxia, in addition to physiological adaptions to hypoxia (3, 4, 5, 6, 7). However, the detailed molecular mechanisms of acute hypoxia adaptation remain largely unknown. In crustaceans, the hypoxia-inducible factor-1 (HIF-1) signaling pathway is involved in hypoxia tolerance (8, 9, 10). Notably, in crustaceans, compensatory changes under hypoxia take place at the hematological level, involving enhancing the affinity of hemocyanin molecules for oxygen and increasing the production of lactate from glucose in hypoxic hemocytes (11, 12, 13, 14). The latter is mainly accomplished by transporting glucose in hemocytes (15). In contrast to mammal’s mature red blood cells, which have no mitochondria and are required to transport oxygen, crustacean hemocytes contain mitochondria and other organelles (16). However, studies in crustaceans suggested that mitochondrial respiration and pyruvate catabolism are suppressed during cellular hypoxia adaptation (17, 18, 19); therefore, further investigation of the molecular mechanisms is required.

The HIF signaling pathway appeared early in metazoan evolution (20), and the HIF-1α, HIF-2α, and HIF-3α have extensive structural homology and have been identified widely, such as in fish (21, 22), mice (23, 24), and humans (25, 26). To date, analogs of HIF-2α and HIF-3α have not been found in crustaceans, and HIF-1α is the master regulator of the cellular response to hypoxia in crustaceans. Interestingly, some crustacean species have lost the HIF-1 pathway, such as the harpacticoid copepod (27). Nevertheless, the HIF pathway appears to be present in decapods (28), and under hypoxic conditions, HIF-1α can induce a switch from oxidative phosphorylation to glycolytic metabolism, which activates the expression of pyruvate dehydrogenase kinase isozyme 1 (PDK1), thereby increasing cell survival (29, 30). Hemocytes are cells that circulate in the hemolymph, and considering that hemocyanins from hemocytes are respiratory proteins, whether oxygen was exhausted in mitochondria of hemocytes before oxygen could reach its destination is unknown; therefore, this metabolic pathway in crustacean hemocytes under hypoxia remains to be characterized.

Under hypoxia, most mammalian cells require glucose as an essential metabolic substrate (31). The facilitated diffusion of glucose across cell membranes is catalyzed by the glucose transporter (GLUT) superfamily, which contains 13 identified members, including GLUT 1 to 12, H(+)-myo-inositol symporter, and four pseudogenes (32). For instance, hypoxia-induced increases in glycolysis are mediated by HIF's induction of GLUTs, such as solute carrier family 2 member 1 (*SLC2A1*, encoding GLUT1) with functions in the blood–brain barrier (33, 34, 35). Importantly, GLUT2 in the liver and intestine regulates the secretion of glucose into the blood (36). Therefore, GLUT1 appears to affect hypoxia signaling reciprocally. Despite the fact that GLUT1 from crustacean is upregulated during hypoxia (15), detailed mechanisms of the transcriptional regulation of *GLUT1* by HIF-1α remain to be fully determined.

In certain types of cells, *de novo* GLUT1 synthesis and its translocation to the plasma membrane are required for increased glucose uptake (37). However, whether HIF-1α-mediated GLUT1 and GLUT2 transporter expression is regulated by hypoxia in crustaceans is unknown. Therefore, the present study aimed to investigate glucose metabolism in primary hemocytes and showed that HIF-1α-induced GLUT1 promotes glucose uptake and lactic acid production under hypoxia. In addition, we demonstrated that GLUT1 is involved in the regulation of cell function in prawns under hypoxia *in vivo* and *in vitro*.

## Results

### Cloning and expression pattern of evolutionarily conserved GLUT1 and GLUT2 in the oriental river prawn (*Macrobrachium nipponense*)

**Figure 1:**
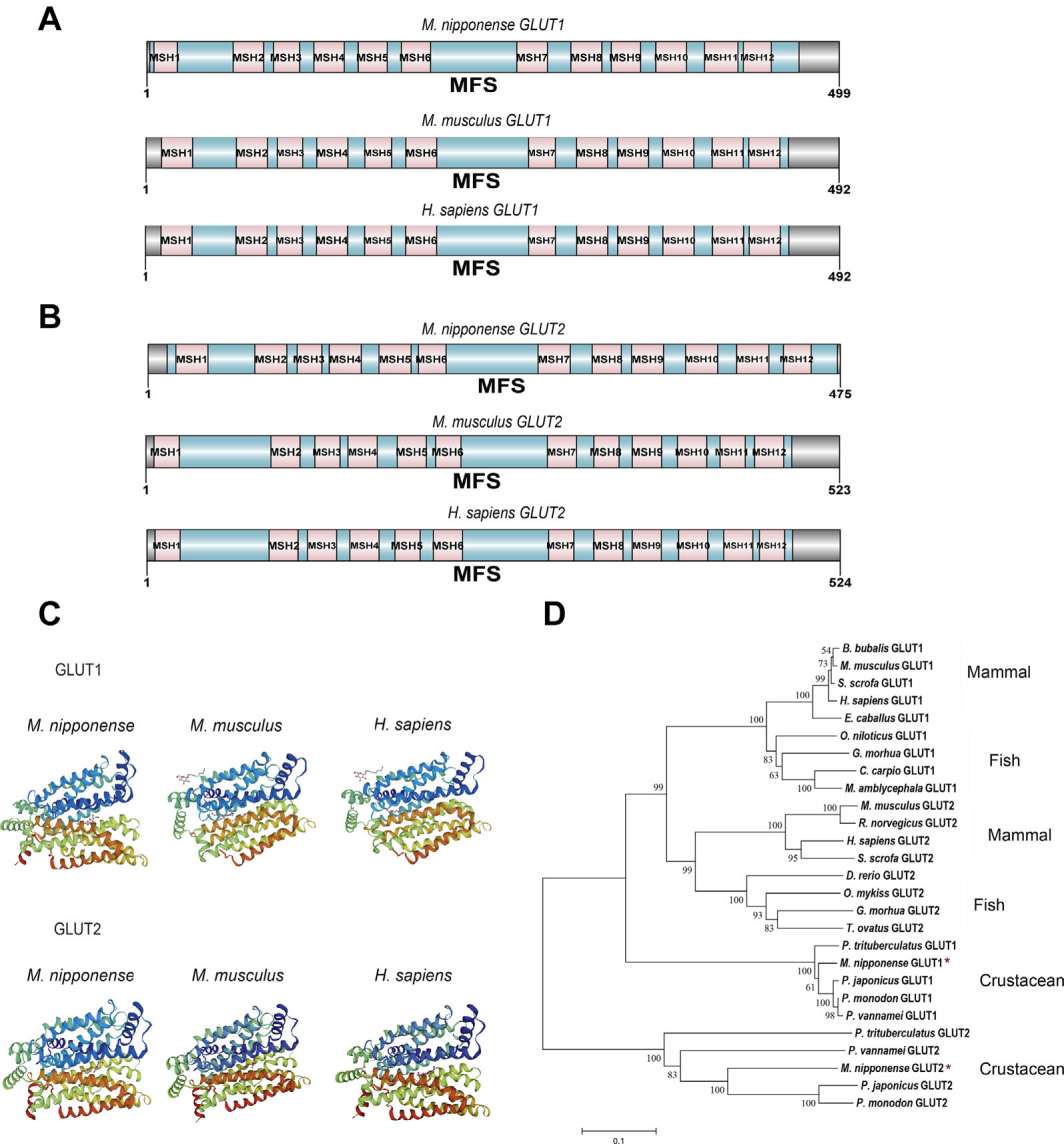
**Evolutionary conservation of GLUT1 and GLUT2 in prawn.***A* and *B*, the domain organization of GLUT1 and GLUT2 in the oriental river prawn (*Macrobrachium nipponense*), mouse (*Mus musculus*), and human (*Homo sapiens*). Twelve conserved transmembrane domains are shown. Nucleotide and deduced amino acid sequence of the prawn *GLUT1* and *GLUT2* cDNAs are shown in Figs. S1 and S2. *C*, prediction of the tertiary structures of prawn (*M. nipponense*), mouse (*Mus musculus*), and human (*Homo sapiens*) GLUT1 and GLUT2 using SWISS-MODEL. *D*, a phylogenetic tree was constructed using the amino acid sequences of GLUT1 and GLUT2 from the indicated species. The phylogenetic tree was constructed using the neighbor-joining algorithm with the MEGA 4.1 program, based on a multiple sequence alignment generated by ClustalW. Bootstrap values of 1000 replicates (percentages) are indicated on the branches. The accession numbers of the selected sequences are listed in Table S1. cDNA, complementary DNA; GLUT, glucose transporter.

The full-length *GLUT1* and *GLUT2* complementary DNAs (cDNAs) were cloned from hemocytes of the oriental river prawn. The *GLUT1* and *GLUT2* cDNAs comprise a short 5′-UTR, open reading frames of 499 and 495 amino acid, respectively, and a relatively long 3′ UTR, and a poly(A) tail (Figs. S1 and S2). *M. nipponense GLUT1* and *GLUT2* cDNA sequences have been submitted to GenBank with accession numbers MT733824 and OM049769, respectively. The oriental river prawn potential transmembrane motifs are evolutionarily conserved, and their domain organization is very similar to that in their mouse and human homologs. The functional transmembrane motifs in the oriental river prawn GLLUT1 protein are organized similarly to those in GLUT2 (Fig. 1, *A* and *B*). Furthermore, the predicted tertiary structures of the oriental river prawn GLUT1 and GLUT2 are well conserved compared with their homologs in mouse and human (Fig. 1*C*). Phylogenetic analysis identified two major branches: vertebrates (mammals and fish) and invertebrates (crustaceans), the latter of which included prawn GLUT1 and GLUT2 (Fig. 1*D*), which agreed with classical zoological systematics. In addition, using multiple sequence alignment, we revealed that the amino acid sequences of oriental river prawn GLUT1 and GLUT2, especially in the transmembrane motifs, are highly conserved compared with those of other crustaceans (Fig. S3). The result showed that oriental river prawn GLUT1 has 85% amino acid identity with GLUT1 of *Litopenaeus vannamei* but 82 and 80% amino acid identity with that of *Penaeus monodon* and *Scylla paramamosain*, respectively. Prawn putative GLUT2 also showed relatively high similarity with GLUT2 from *P. monodon* (68%).

**Figure 2:**
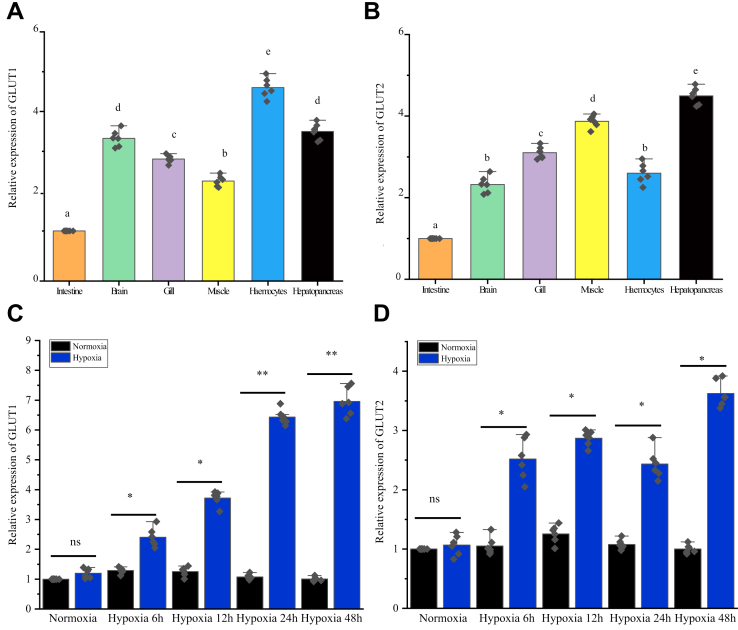
**Tissue distribution and mRNA expression profiles of *GLUT1* and *GLUT2* in oriental river prawns.***A* and *B*, the tissue distribution of *GLUT1* and *GLUT2* mRNA. RNA samples were extracted from healthy prawns, and *GLUT1* and *GLUT2* expression was studied using qRT–PCR (with β-actin as the internal reference gene). Shown are the means ± standard error (SE; n = 6). Different *lowercase letters* indicate the significance (determined using one-way ANOVA). *C* and *D*, qRT–PCR analysis of the mRNA expression profiles of *GLUT1* and *GLUT2* in hemocytes under hypoxia (1% O_2_), using β-actin as the reference gene. Shown are the means ± SE (n = 6). ∗∗*p* < 0.01, ∗*p* < 0.05 (Student’s *t* test). GLUT, glucose transporter; qRT–PCR, quantitative RT–PCR.

We identified *GLUT1* and *GLUT2* expression in tissues that harbor hemocytes (Fig. 2, *A* and *B*), suggesting a role for their encoded proteins in prawn physiology. To identify whether hypoxia could induce *GLUT1* and *GLUT2* expression, DO at 1.8 ± 0.2 mg/l was used to simulate hypoxia *in vivo*. Significantly higher expression levels of *GLUT1* and *GLUT2* were observed in prawns under hypoxia for 24 h (Fig. 2, *C* and *D*), thus demonstrating its potential participation in the response to hypoxic stress.

### HIF-1 mediates the induction of GLUT1 transcription in response to hypoxia

**Figure 3:**
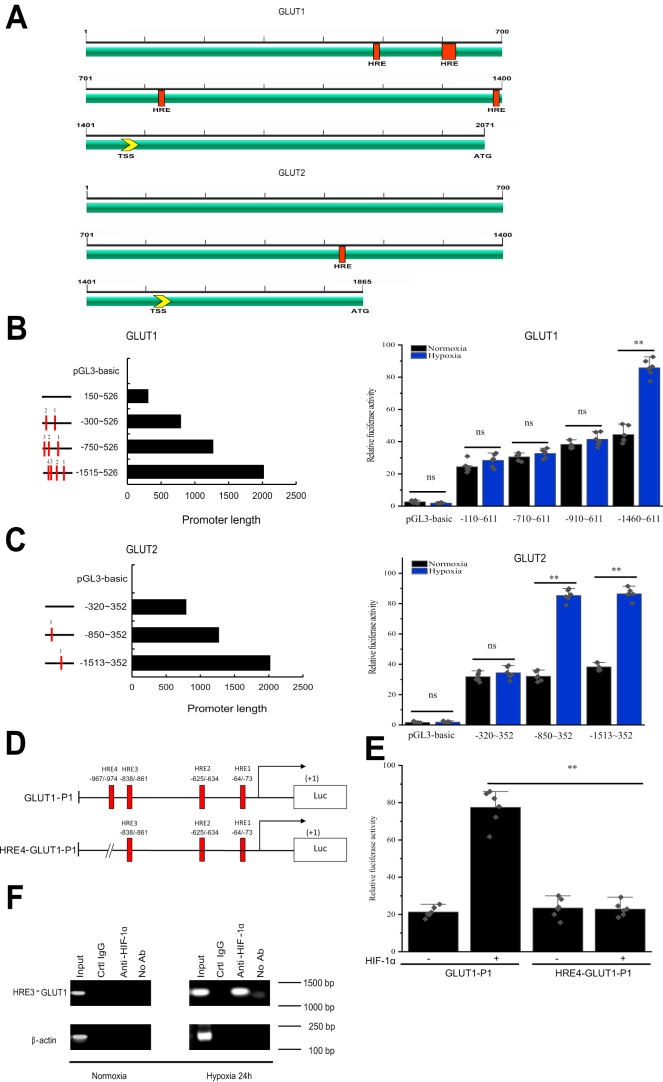
**The preferentially induction of GLUT1 expression *via* an HIF-1α binding site under hypoxia.***A*, a schematic diagram of the *GLUT1* and *GLUT2* gene promoter region. Putative transcription factor–binding sites are *underlined* and labeled according to the TRANSFAC database (http://gene-regulation.com/); the nucleotide sequence of the prawn *GLUT1* and *GLUT2* gene 5′ flanking region is shown in Figs. S3 and S4. *B*, the expression of *GLUT1* and *GLUT2* promoter constructs in transiently transected hemocytes. An illustration of the promoter-luciferase reporter constructs is shown on the *left*. The locations of the potential regulatory elements, HREs, are indicated using *red lines*. *C*, the luciferase activity of each construct relative to that of the empty vector (pGL3-basic) in transiently transfected hemocytes. The data are expressed as fold induction relative to the empty vector, and the error bars represent the mean ± standard error (SE) of six replicate trials (n = 6), ∗∗*p* < 0.01 (Student’s *t* test). *D*, a schematic representation of the *GLUT1* promoter mutants. ΔHRE4-GLUT1-P1 is a mutated *GLUT1* promoter at putative HRE4. *E*, hemocytes were transiently transfected with each promoter construct along with an HIF-1α expression plasmid. Values represent the means ± SE of six independent experiments (n = 6), ∗∗*p* < 0.01 (Student’s *t* test). *F*, HIF-1α binds to HRE4 in the *GLUT1* promoter under hypoxic conditions in prawn hemocytes as determined by ChIP assays. ChIP, chromatin immunoprecipitation; GLUT, glucose transporter; HIF, hypoxia-inducible factor; HRE, hypoxia-response element; TSS, transcription start site.

We examined the 5′-flanking sequence (2071 and 1865 bp) of the two *GLUT1* and *GLUT2* gene loci of the oriental river prawn genome using genome walking (Figs. S4 and S5), and these regions were predicted to contain multiple hypoxia-response elements (HREs) (Fig. 3*A*). We next tested the potential roles of these *GLUT1* and *GLUT2* promoter HREs in hypoxia-related gene regulation by generating a collection of *GLUT1* and *GLUT2* promoter fragments, which were placed 5′ of a luciferase reporter gene (Fig. 3*B*). Following transfection of these constructs into hemocytes and incubation under normoxia, incubation of the pGL3-*GLUT1*– and pGL3-*GLUT2*–expressing cells under hypoxia for 24 h resulted in significantly enhanced luciferase activity in comparison to that under normoxic conditions. Among the four HREs, constructs containing first three HREs (−1460 to 611) showed decreased luciferase activity compared with that from the full-length promoter under hypoxic conditions (*p* < 0.01), suggesting that the last HRE of the *GLUT1* promoter is involved in transcriptional activation under hypoxic conditions (Fig. 3*C*). Next, we constructed HRE4–RGN-P1, which contained a 7 bp deletion of HRE4 (−967 to −974) (Fig. 3*D*), which was used to identify the function of HRE in the activity of the *GLUT1* promoter. Transient transfection analyses showed that deletion of the presumptive HRE4 site decreased the HIF-1α-mediated activation of the *GLUT1* promoter significantly (Fig. 3*E*). Collectively, these results showed that HRE4 is important for HIF-1α binding and activation of the *GLUT1* promoter. Furthermore, chromatin immunoprecipitation (ChIP) assays showed that HRE4 is directly involved in hypoxia-induced *GLUT1* transcriptional activation (Fig. 3*F*).

### Glucose metabolism in prawn primary hemocytes under hypoxic conditions

**Figure 4:**
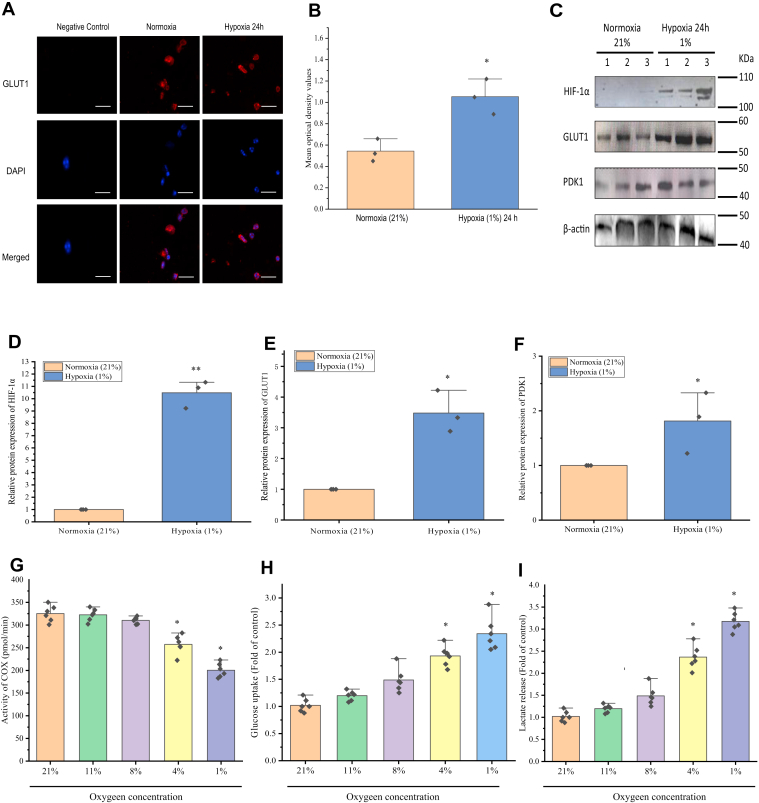
**Involvement of HIF-1α–GLUT1 in the glycolytic reprogramming of primary hemocytes during hypoxia.***A*, immunofluorescence of GLUT1 in the hemocytes of oriental river prawns under hypoxia for 24 h. Confocal microscopy: fluorescence was developed using secondary antibodies conjugated with Alexa 568 (*red*). Nuclei were stained with 4′,6-diamidino-2-phenylindole (DAPI) (*blue*). In the negative control, primary antibodies were replaced with normal nonimmune serum. The *white line* in the *lower right corner* represents the scale bar (50 μm). Immunofluorescence of GLUT2 in hemocytes is shown in Fig. S5. *B*, the mean absorbance values used for quantification of the cell membrane expression of GLUT1 in response to hypoxia 24 h. All values are presented as the mean ± standard error (SE) (n = 3), ∗*p* < 0.05 (Student’s *t* test). *C*, HIF-1α, GLUT1, and PDK1 protein levels in hemocytes under hypoxia for 12 h were determined using Western blotting analysis. β-actin was used as an internal control. *D*–*F*, the quantitative data for the protein levels in prawn hemocytes under hypoxia for 24 h. ImageJ was used for densitometry quantification of the immunoreactive protein bands on the Western blot. The error bars represent the mean ± SE of three replicate trials (n = 3). ∗∗*p* < 0.01, ∗*p* < 0.05 (Student’s *t* test). *G*, the cytochrome *c* oxidase activity of hemocytes was calculated in the presence of ascorbate (100 mM) and TMPD (1 mM; 21% O_2_, 4 h—11% O_2_, 4 h—8% O_2_, 4 h—4% O_2_, 4 h—1% O_2_). *H* and *I*, the effect of hypoxia on ^3^H-2-DOG glucose uptake and lactate secretion in hemocytes under hypoxia for 24 h. The abilities of glucose uptake and lactate secretion were expressed as a ratio of the hypoxia treatment sample in the control normoxia sample. The error bars represent the mean ± standard error (SE) of six replicate trials (n = 6). Student’s *t* test was performed to calculate the significant differences. ∗*p* < 0.05 represents control (21% O_2_) *versus* 11%, 8%, 4%, and 1% O_2_ concentration, respectively. Glycolysis-related gene expression levels and mitochondrial complex I−IV activities in hemocytes of prawns under hypoxia are shown in Fig. S6, illustrating that hypoxia induced glycolytic remodeling of hemocytes in crustaceans. GLUT, glucose transporter; ^3^H-2-DOG, ^3^H-2-deoxy-d-glucose; HIF, hypoxia-inducible factor; PDK1, pyruvate dehydrogenase kinase isozyme 1; TMPD, tetramethyl-*p*-phenylenediamine.

GLUT1 is a GLUT that exists widely in various organisms (36). We next evaluated whether hypoxia upregulates GLUT1 preferentially, using immunofluorescence. The results showed that GLUT1 could be detected in the hemocyte cell membrane with a strong fluorescence signal after hypoxia for 12 h (Fig. 4*A*), indicating that GLUT1 is induced and accumulates in the cell membrane under hypoxia. There was no significant GLUT2 fluorescence signal in the cell membrane of hemocytes under normoxia compared with that under hypoxia for 12 h (Fig. S6), suggesting that hypoxia upregulates GLUT1 *via* HIF-1α preferentially in hemocytes. In fact, hypoxia rapidly caused GLUT1 translocation to cell membrane in hemocytes in response to hypoxia for 12 h but not that of GLUT2 (Fig. 4*B*). Furthermore, the levels of HIF-1α and GLUT1 in hemocytes after hypoxia for 12 h were evaluated using Western blotting (Fig. 4*C*). The levels of HIF-1α and GLUT1 were significantly (*p* < 0.05) increased in hemocytes in response to hypoxia for 12 h (1% O_2_) (Fig. 4, *E* and *F*). The results showed a hypoxia time-dependent accumulation of HIF-1α and GLUT1. PDK phosphorylates and inhibits pyruvate dehydrogenase, which is a rate-limiting enzyme that regulates the entry of pyruvate into the tricarboxylic acid (TCA) cycle. We also found that PDK1 levels were increased significantly (*p* < 0.01) after hypoxia for 12 h (Fig. 4*G*), implying that under hypoxic conditions, the HIF-1α–GLUT1–PDK1 axis causes glycolytic reprograming by reducing pyruvate flux into the TCA cycle. In addition, hypoxia induced glycolysis in prawn hemocytes *in vivo* (Fig. S7).

To monitor the glucose oxidative phosphorylation ability of primary hemocytes after hypoxia for 12 h, the activity of hemocyte cytochrome *c* oxidase was measured in the presence of ascorbate and tetramethyl-*p*-phenylenediamine, which ensures an adequate supply of electrons for cytochrome *c* oxidase. Cytochrome *c* oxidase activity decreased at O_2_ concentrations of 1 to 4% but was unchanged under moderate hypoxia (8% oxygen) (Fig. 4*H*), which further supported our observation that maintenance of hemocyte glucose oxidation requires 8% O_2_. Accordingly, glucose uptake in hemocytes was significantly increased by severe hypoxia (1–4% O_2_) for 24 h compared with that in the normoxia control (21% O_2_). This increase was consistent with lactate production (Fig. 4, *I* and *J*), suggesting that glucose is necessary in hemocytes to supply energy in response to hypoxia. Moreover, our findings indicated that GLUT1 contributes to relieving hypoxic stress in hemocytes by regulating their glucose uptake.

### HIF-1α has a vital function in the glycolytic activity of primary hemocytes

**Figure 5:**
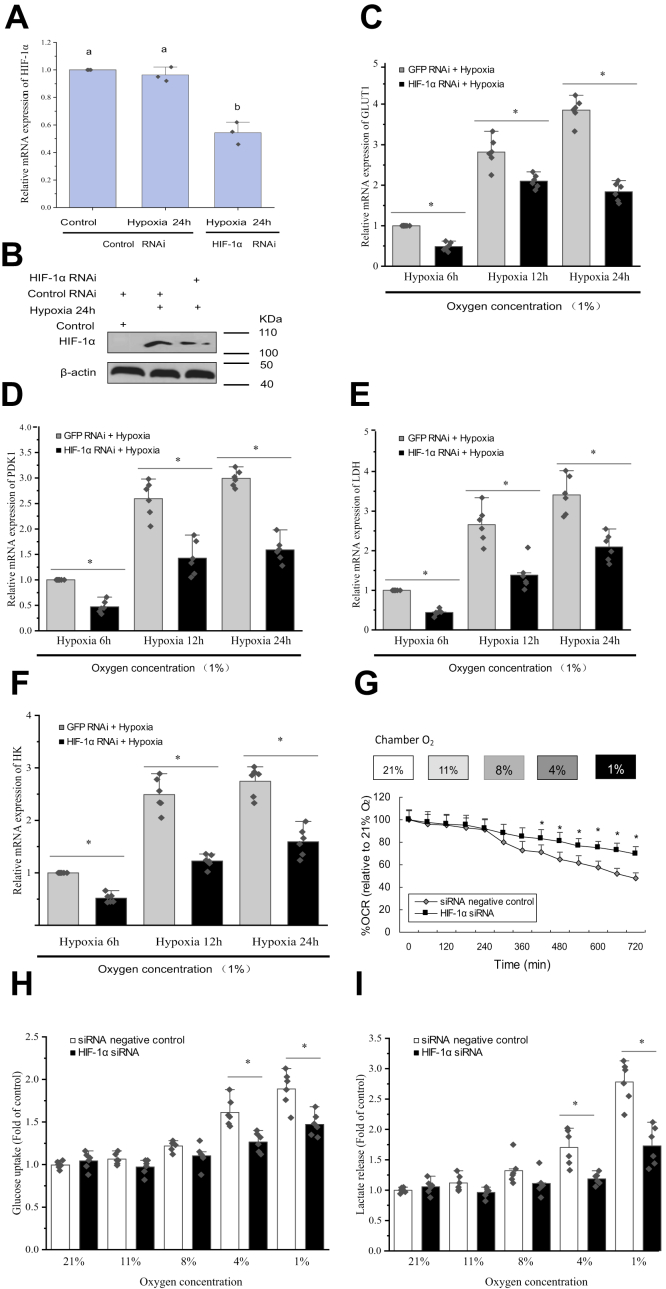
**HIF-1α-dependent glycolytic reprogramming in primary hemocytes during hypoxia.***A*, the effects of RNAi on HIF-1α. An *HIF1α*siRNA (siHIF-1α) was designed to knockdown HIF-1α expression, and HIF-1α expression in prawn hemocytes was determined 24 h posthypoxia with siHIF-1α by qRT–PCR (with siGFP as a control). Shown are the means ± standard error (SE; n = 3). Three independent repeats were performed with different *lowercase letters* indicating the significance (one-way ANOVA). *B*, hemocytes were knocked down for HIF-1α, which was confirmed using immunoblotting. *C*–*F*, knockdown of HIF-1α decreased the hypoxic induction of glucose transporter 1 (GLUT1), pyruvate dehydrogenase kinase isoenzyme 1 (PDK1), hexokinase (HK), and lactate dehydrogenase (LDH) in hemocytes (with siGFP as a control). *G*, the cellular oxygen consumption rate (OCR) of hemocytes under hypoxia (3 h—11% O_2_, 3 h–8% O_2_, 3 h–5% O_2_, and 3 h–1% O_2_) was plotted as percent of the OCR in 21% O_2_ (*y*-axis) *versus* time (*x*-axis). *H* and *I*, the glucose uptake by hemocytes was examined using a 2-deoxyglucose (2-DG) uptake assay. Lactate was measured using a lactate assay kit. The glucose uptake and lactate secretion abilities were expressed as a ratio of the hypoxia 24 h treatment sample in the control normoxia sample. The error bars represent the mean ± SE of six replicate trials (n = 6). ∗*p* < 0.05 (Student’s *t* test), which marks significant differences in the control RNAi *versus* HIF-1α RNAi. The critical role of HIF-1α in glycolytic reprogramming in hypoxia was also observed in *Drosophila* S2 cells (Fig. S7); supporting the notion that HIF-1*α* plays an essential role in the regulation of mitochondrial activity in invertebrates. GLUT1, glucose transporter 1; HIF-1α, hypoxia-inducible factor-1α; qRT–PCR, quantitative RT–PCR.

Next, we tested the involvement of HIF-1α in the hypoxic *GLUT1*, *GLUT2*, and *PDK1* induction using siRNA-mediated knockdown of HIF-1α expression in hemocytes under hypoxia for 12 h. Specific siRNA targeting the transcripts of HIF-1α was designed to silence HIF-1α expression. Successful knockdown of HIF-1α was confirmed using quantitative RT–PCR (qRT–PCR), indicating about 50% reduction in HIF-1α transcript levels in the silenced primary hemocytes (Fig. 5*A*), HIF-1α knockdown suppressed the hypoxia-induced increase in the HIF-1α protein level (Fig. 5*B*). Knockdown of HIF-1α significantly (*p* < 0.05) reduced the hypoxia-induced mRNA expression of *GLUT1* and *PDK1* (Fig. 5, *C* and *D*), experimentally confirming that HIF-1α mediates the observed hypoxic induction of *GLUT1* and *PDK1* transcription. These results support the view that HIF-1α physically associates with the *GLUT1* promoter by binding to HRE4 and activates *GLUT1* transcription, establishing that in addition to *PDK1*, *GLUT1* is a direct transcriptional target of HIF-1α in prawns in response to hypoxia. Meanwhile, hypoxia also induced the expression of glycolysis-related genes (*HK* [encoding hexokinase] and *LDH* [encoding lactate dehydrogenase]). Knockdown of HIF-1α significantly (*p* < 0.05) reduced the hypoxic induction of *HK* and *LDH* (Fig. 5, *E* and *F*).

The oxygen consumption rate (OCR), with glucose as the energy substrate, was determined in cells to assess mitochondrial-dependent glucose oxidation. We monitored the OCR under normoxic conditions (21% oxygen) and then continued the measurements every 3 h as the cells were subjected to decreasing oxygen concentrations between time points. The OCR in hemocytes decreased significantly (*p* < 0.05) at 1 to 4% O_2_, suggesting that to maintain hemocyte glucose oxidation, approximately 50% of oxygen concentration was required (Fig. 5*G*). The OCR was determined in HIF-1α knockdown cells to investigate the function of HIF-1α in glycolytic reprogramming. HIF-1α knockdown markedly reversed the decrease in the OCR under hypoxia (Fig. 5*G*). The vital function of HIF-1α in glycolytic reprogramming under hypoxia was confirmed in *Drosophila* S2 cells (Fig. S8), which supported the hypothesis that the function of HIF-1α is vital to support the OCR. Although glucose uptake was significantly (*p* < 0.05) inhibited, glucose uptake and lactate output were not enhanced by HIF-1α knockdown, indicating that in cells knocked down for HIF-1α, glycolysis was inhibited in the presence of 1 to 4% O_2_ (Fig. 5, *H* and *I*).

### The participation of GLUT1 in glucose uptake and cell function under hypoxia *in vitro*

**Figure 6:**
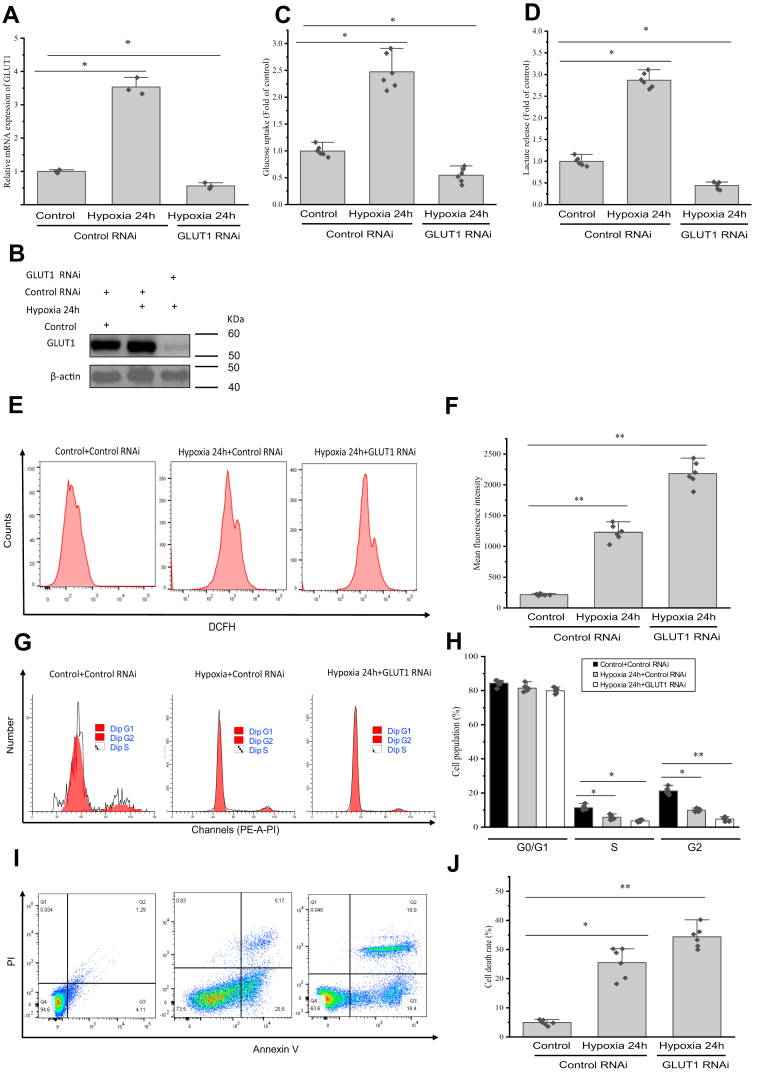
**Involvement of GLUT1 in glucose uptake and reactive oxygen species (ROS) production in primary hemocytes during hypoxia.***A*, hemocytes were transfected with an siRNA targeted against *GLUT1* or with a nontargeting siRNA as a control. The error bars represent the mean ± standard error (SE) of three replicate trials (n = 3), ∗*p* < 0.05 (Student’s *t* test). Forty-eight hours after siRNA transfection, hemocytes were treated with or without hypoxia for 24 h. *B*, hemocytes were knocked down for *GLUT1*, which was confirmed using immunoblotting. *C* and *D*, the effects of hypoxia on glucose uptake and lactate production in hemocytes were examined and expressed as a ratio of the normoxia treatment sample in the control siRNA sample. *E*, intracellular ROS in hemocytes treated with hypoxia or *GLUT1* knockdown were detected using flow cytometry. *F*, summarized data showing that *GLUT1* knockdown aggravated the effect of hypoxia-induced ROS generation of prawn hemocytes. *G*, flow cytometry analysis evaluating the influence of hypoxia or *GLUT1* knockdown on the cell cycle in hemocytes. *H*, summarized data showing that *GLUT1* knockdown aggravated the effect of the hypoxia-inhibited cell cycle in hemocytes. *I*, the effect of hypoxia or *GLUT1* knockdown on cell apoptosis was determined using flow cytometry. *J*, summarized data showing that *GLUT1* knockdown aggravated the effect of hypoxia-induced cell apoptosis in hemocytes. The error bars represent the mean ± SE of six replicate trials (n = 6). ∗∗*p* < 0.01 and ∗*p* < 0.05 (Student’s *t* test), which mark significant differences between the normoxia treatment sample in the control siRNA sample and the hypoxia groups subjected to control or *GLUT1* siRNA. GLUT1, glucose transporter 1.

To determine if GLUT1 mediates glucose uptake under hypoxia, we knocked down *GLUT1* in hemocytes. *GLUT1* knockdown suppressed the hypoxia-induced increase in the GLUT1 protein level (Fig. 6, *A* and *B*). Hypoxia increased glucose uptake and lactate production, which was abrogated after *GLUT1* knockdown (Fig. 6, *C* and *D*). Subsequently, we evaluated whether *GLUT1* knockdown affected the hypoxia-induced changes in intracellular reactive oxygen species (ROS) in hemocytes. The results showed that hypoxia significantly enhanced intracellular ROS production compared with that in the control group, and this increase was significantly (*p* < 0.01) enhanced by *GLUT1* knockdown *in vitro* (Fig. 6, *E* and *F*). According to flow cytometry analysis, *GLUT1* knockdown disturbed the hemocyte cell cycle. Specifically, hemocyte entry from the gap 0/gap 1 to the synthesis phases was accelerated, and gap 2 phase was significantly (*p* < 0.05) shortened after *GLUT1* knockdown (Fig. 6, *G* and *H*). In addition, a flow cytometry assay showed that the extent of hypoxia-induced apoptosis in hemocytes was significantly increased (*p* < 0.05) by *GLUT1* knockout *in vitro* compared with that in the scrambled control group exposed to hypoxia (Fig. 6, *I* and *J*).

### *In vivo* participation of GLUT1 in glucose uptake and cell function during hypoxia

**Figure 7:**
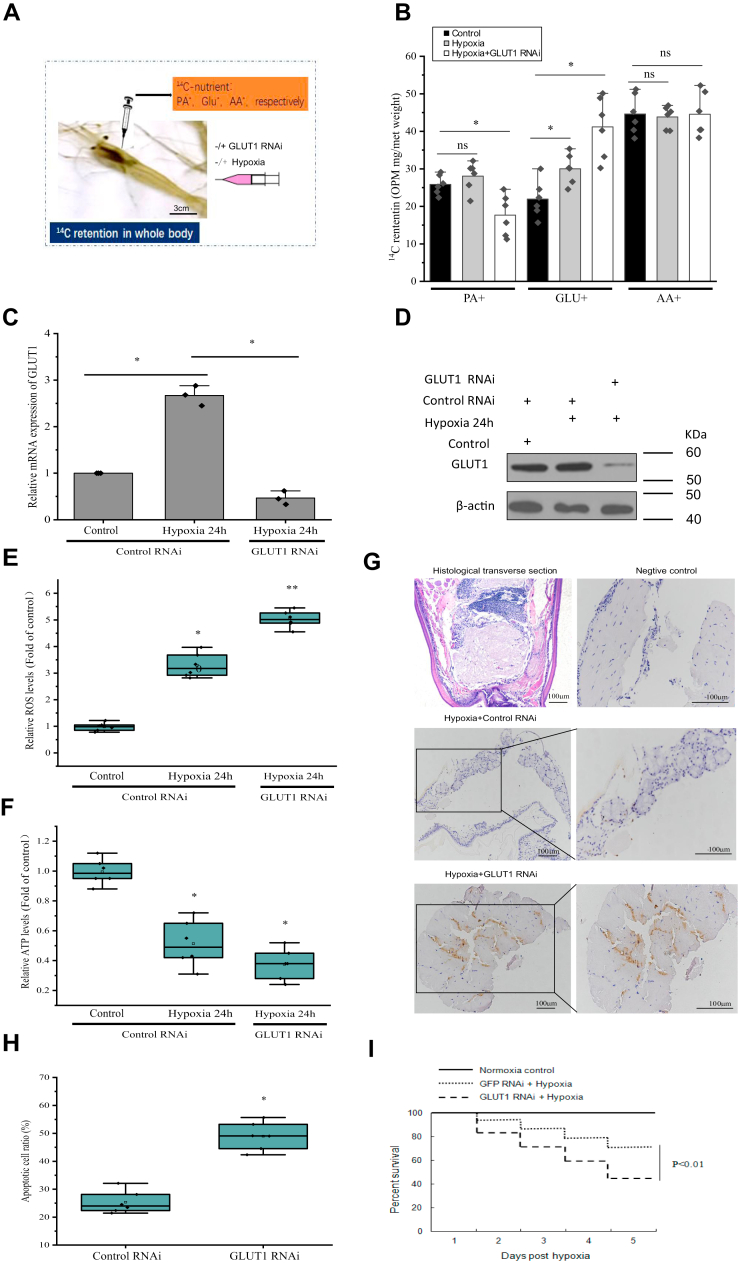
**Involvement of GLUT1 in the regulation of brain cell function of prawns in response to hypoxia *in vivo*.***A*, schematic diagram of ^14^C-labeled nutrient tracking test in prawns injected with 10 μg of siGLUT1 (with siGFP as a control) after 24 h. *B*, ^14^C-retention in the whole body at 2 h after the injection of PA^∗^ or Glu^∗^, or AA^∗^ in siGFP and siGLUT1 knockdown prawns (n = 6), ∗*p* < 0.05 (Student’s *t* test). *C*, prawns were each injected with 10 μg of siGLUT1 (with siGFP as a control), and *GLUT1* expression in prawn hemocytes was determined at 24 h posthypoxia using qRT–PCR. The error bars represent the mean ± standard error (SE) of three replicate trials (n = 3), ∗*p* < 0.05 (Student’s *t* test). *D*, hemocytes were knocked down for *GLUT1 in vivo*, which was also confirmed using immunoblotting. *E* and *F*, intracellular reactive oxygen species (ROS) and ATP levels in *GLUT1*-silenced prawn under hypoxia. Prawns were each injected with 10 μg of siGLUT1 (with siGFP as a control), and intracellular ROS and ATP levels in the prawn brains were determined at 24 h after injection with siGLUT1. Values are normalized to those of normoxic conditions. The error bars represent mean ± SE of six replicate trials (n = 6). ∗∗*p* < 0.01, ∗*p* < 0.05 (Student’s *t* test), which mark significant differences between the normoxia and the hypoxia groups subjected to *GLUT1* or control siRNA. *G*, knockdown of *GLUT1* exacerbates hypoxia-induced apoptosis in adult prawn brains. TUNEL staining of the brains of GLUT1-silenced prawns in response to hypoxia for 24 h. Apoptotic cells are colored *gray*; healthy cells are colored *baby blue*. The *black line* in the *lower right corner* represents the scale bar (100 or 50 μm). *H*, the ratio of apoptotic brain cells to healthy brain cells in *GLUT1*-silenced prawns. The error bars represent ± SE of six replicate trials (n = 6). ∗*p* < 0.05 (Student’s *t* test), which marks significant differences between the hypoxia treatment sample in the control siRNA sample and the hypoxia groups subjected to control or *GLUT1* siRNA. *I*, knockdown of GLUT1 aggravated prawn hypoxic death. Prawns were each injected with 10 μg of siGLUT1 (with siGFP as a control), and their 5-day survival posthypoxia (1.8 mg/l) was assessed. The error bars represent ±SE of triplicate trials (n = 3). ∗∗*p* < 0.01 (Student’s *t* test), which marks significant differences between the hypoxia treatment sample in the control siRNA sample and the hypoxia groups subjected to control or *GLUT1* siRNA. GLUT1, glucose transporter 1; qRT–PCR, quantitative RT–PCR.

To assess the role of GLUT1 in the overall nutrient metabolism regulation *in vivo*, the use of [1-^14^C]-palmitic acid (PA^∗^), d-[1-^14^C]-Glu (Glu^∗^), and l-[^14^C(U)]-amino acid (AA^∗^) were tracked after their individual intraperitoneal injection (Fig. 7*A*). The radioactivity retained in the bodies of prawns injected with PA^∗^ or AA^∗^ was significantly (*p* < 0.05) lower in the *GLUT1* knockdown group, whereas the radioactivity retained after Glu^∗^ injection was significantly (*p* < 0.05) higher than that of the control normoxia group (Fig. 7*B*). To examine whether the production of ROS and ATP during hypoxia is mediated by GLUT1, *GLUT1* was knocked down by injection of siRNA. Hypoxia-increased GLUT1 protein expression was suppressed by GLUT1 siRNA treatment (Fig. 7, *C* and *D*). We hypothesized that under hypoxia, a lack of attenuation of flux through the TCA cycle would result in ineffective electron transfer in the mitochondria, causing increased production of ROS (37, 38). In deed, hypoxia resulted in increased intracellular ROS levels in *GLUT1* knockdown prawns compared with those in the scrambled control siRNA group under hypoxia (Fig. 7*E*).

In *GLUT1* knockdown prawns, the ATP level was lower under hypoxia compared with that in the scrambled control siRNA group under hypoxia (Fig. 7*F*). This reduction might have resulted from attenuated ATP production or enhanced ATP consumption by mitochondria to maintain the mitochondrial membrane potential. In turn, enhanced ROS levels might trigger apoptosis (37). Therefore, we counted the number of apoptotic brain cells of *GLUT1* knockdown adult prawns and in the scrambled control siRNA under hypoxia using TUNEL assays. The brains of the *GLUT1* knockdown prawns contained significantly more apoptotic cells than that of the control siRNA group after 12 h of hypoxia (Fig. 7, *G* and *H*). These data indicated that inhibiting *GLUT1* expression promotes the apoptosis induced by hypoxia. Finally, *GLUT1* knockdown in prawns greatly reduced their survival rate upon challenge with hypoxia (Fig. 7*I*).

## Discussion

In invertebrate research, the essential role of HIF-1α signaling in the hypoxic response has been reported (39, 40). The mechanisms of the hypoxic stress response in different tissues and cells have been evaluated in *Drosophila*, such as in adipose tissue (41), brain tissue (42), and blood cells (43). Research in *Drosophila* emphasized that the HIF-1 pathway is functionally conserved, suggesting its ancient evolutionary origin. In the past decade, members of GLUT protein family have been identified, which appear to be highly conserved across evolutionary history, including their 12 conserved transmembrane domains. GLUT genes have been cloned and widely confirmed in invertebrates, such as GLUT1 (15), GLUT2 (44), and GLUT4 (45). Herein, our discovery of the key function of GLUT1 in oriental river prawn expands the current knowledge of crustacean hypoxic stress, and only GLUT1 (but not GLUT2) significantly translocates glucose in prawn hemocytes *in vitro*. Hypoxia increased glucose uptake and lactic acid production in prawn hemocytes *via* the HIF-1α–GLUT1–PDK1 pathway; and the essential role of GLUT1 in the prawn blood–brain barrier during hypoxia was revealed. Our experimental evidence suggests that GLUT1 upregulation by HIF-1α might be involved in hypoxia-mediated glycolysis regulation and is required to import large amounts of extracellular glucose to promote glycolysis and produce energy in hemocytes.

GLUT1 undertakes basal glucose uptake and storage rather than insulin-dependent glucose uptake (36) and is particularly abundant in erythrocytes. This was similar to our results for the tissue expression profile of GLUT1, which was demonstrated to be ubiquitously expressed and detected in all the tissues analyzed, but with the highest expression level in hemocytes among the five tissues examined. A reasonable explanation was that the various isoforms have a tissue-specific expression profile and are responsible for glucose homeostasis in crustaceans in response to hypoxia. To date, the insulin signaling pathway has been well described in the fruit fly *Drosophila melanogaster* (46). Although the insulin signaling pathway remains largely unexplored in nonmodel invertebrates, our draft genome sequence assembly showed a number of insulin-like peptides in *M. nipponense* compared with those in other invertebrates and demonstrated the presence of multiple insulin-like receptors (47). Thus, we first investigated if hypoxia significantly induced *GLUT1* and *GLUT2* mRNA in hemocytes. The result showed that the relative *GLUT2* mRNA level in hemocytes was higher under hypoxia; however, at the protein level, only the level of GLUT1 was higher in prawn hemocytes under hypoxia, suggesting that GLUT1 upregulation is essential for the response to hypoxia stress in hemocytes and the subsequent energy supply.

Hypoxia was reported to activate glycolysis in the hepatopancreas and brain of prawns (48, 49). In addition, Gene Ontology analysis of transcriptome data (50) showed enrichment of terms related to glycolysis among hypoxia-upregulated genes. Therefore, we speculated that the hypoxia-induced increase in glucose uptake is associated with glycolysis activation. The function of active glucose uptake under hypoxia is unknown. Therefore, which glucose metabolic pathways are activated and their involvement in hypoxia in hemocytes should be determined. Mammalian red blood cells are small anucleate cells that have a critical role as suppliers of oxygen to all regions of the body (44). Compared with mammalian red blood cells, hemocytes not only contain hemocyanin as oxygen carrier but also have an equally important role as regulators of oxygen distribution through an especially open circulatory system in crustaceans. Thus, it was reasonable to speculate that hemocytes could be involved in the regulation of cellular metabolism. Interestingly, although a functioning mitochondrial electron transport chain is required for glucose oxidation, in hypoxic hemocytes containing mitochondria, cytochrome *c* oxidase activity is suppressed, producing a metabolic shift toward classical glycolysis. HIF-1α-induced glycolysis activation was reported to have a beneficial effect on ATP synthesis under severely hypoxic conditions (51). Herein, the HIF-1α–PDK1 axis actively modulated glucose metabolism from glucose oxidation to glycolysis, resulting in decreased cellular ATP levels, and demonstrating the dual role of HIF-1α in ATP synthesis. GLUT1 induction by HIF-1α promotes glucose metabolite flux from the mitochondria to glycolysis, implying that it might be a common feature of hemocyte glycolysis to improve survival under severe hypoxia. Similar to our results for the effect of *GLUT1* knockdown on ROS production and apoptosis, some reports showed that these effects enhanced cell survival, and decreased ROS levels were caused by increased glucose uptake and increased plasma membrane GLUT-1 expression (52, 53, 54).

Our previous studies in the oriental river prawn confirmed that the glycolytic pathway is regulated by HIF-1α *via* transcriptional activation of glycolytic enzyme genes in prawn muscle tissue in response to hypoxia, including LDH and HK (55, 56). Furthermore, the present study confirmed that the *GLUT1* promoter contains an HIF-1α-binding HRE using ChIP. We revealed that inhibiting the binding of HIF-1α to the HRE decreased *GLUT1* mRNA expression under hypoxia. This suggested that GLUT1 function *via* HIF-1α in crustaceans, as they do in mammals (33). Herein, we observed that GLUT1 upregulation is accompanied by increased HIF-1α levels. GLUT1 is responsible for basal glucose uptake, being independent of insulin and has a high affinity for glucose (57). However, so far, no study has investigated the effect of *GLUT1* knockdown on glucose uptake and lactic acid production in crustaceans to confirm this evolutionarily conserved pathway. Consequently, our results clearly showed that knockdown of *GLUT1* reversed the hypoxia-mediated increase in glucose uptake, suggesting a major role for this transporter in glucose uptake. Consistently, *GLUT1* knockdown in adipocytes and stromal cells reduced glucose uptake by about 50% (58, 59). GLUT1 is expressed widely in all tissues but shows its highest expression in erythrocytes and endothelial cells of the adult blood–brain barrier (60). However, it is believed to participate in minimal glucose uptake in all cells. GLUT1-deficiency syndrome results in metabolic encephalopathy caused by a lack of glucose uptake by the central nervous system (61). In prawn hemocytes during hypoxia *in vitro*, inhibition of GLUT1 prevents the uptake of glucose by inducing cell toxicity and apoptosis. Importantly, *GLUT1* knockdown *in vivo* also significantly increased hypoxia-mediated brain apoptosis. Indeed, the increased apoptosis in the brain tissue of *GLUT1* knockdown prawns under hypoxia was related to GLUT1-specific transport of glucose across the blood–brain barrier (62, 63). Crustacean GLUT1 seems to be the main carrier in blood-tissue barriers of hemocytes. GLUT2 functions as a high-capacity transporter, permitting constitutive glucose flux into or out of the cells (64). During hypoxia, GLUT2 possibly functions mainly in the hepatopancreas, where it transports glucose into or out of hemocytes, strongly suggesting that hypoxia promotes the increased binding of HIF-1α to the *GLUT2* promoter region. Hypoxia regulates two of the three known GLUTs in prawns; however, their expression profiles are different. These results imply that in crustaceans, the two GLUTs participate in counteracting the effects of hypoxia; however, it is possible that other, as yet uncharacterized, isoforms are also involved.

**Figure 8:**
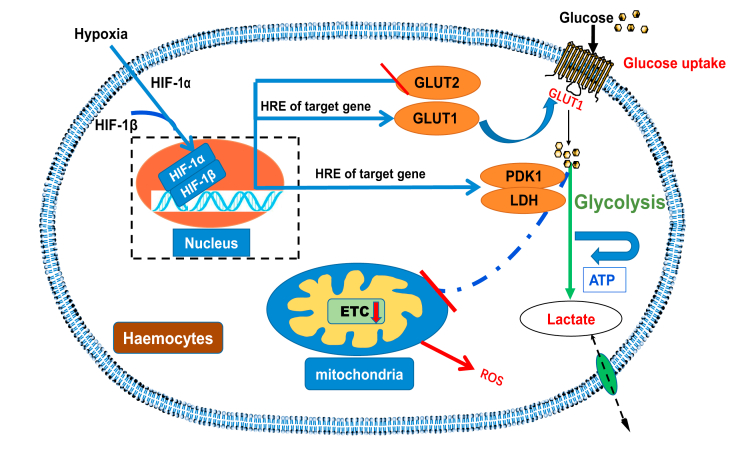
**The schematic representation of HIF-1α–GLUT1-mediated hypoxia regulation of glucose metabolism and cell function.** ETC, electron transfer chain; GLUT, glucose transporter; HIF-1α, hypoxia-inducible factor-1α; HRE, hypoxia response element; LDH, lactate dehydrogenase; PDK1, pyruvate dehydrogenase kinase isozyme 1; ROS, reactive oxygen species.

The present study comprises a conceptual model demonstrating HIF-1α–GLUT1-induced glycolysis regulation in hypoxic hemocytes (Fig. 8). Our findings also revealed a connection between intracellular ROS production and hypoxia-induced metabolic reprogramming. Consequently, we suggest that in hemocytes, GLUT1 ensures increased glucose uptake during hypoxia, which is typically accompanied by increased HIF-1α levels and activated glycolysis, whereas GLUT2 functions in the hepatopancreas to stimulate the release of ingested glucose to hemocytes.

## Experimental procedures

### Animals and primary cultured hemocytes

The Committee on the Use of Live Animals in Teaching and Research of the Shanghai Ocean University approved all the prawn experiments. The Qingpu aquatic farm provided healthy oriental river prawns, which were transported rapidly to the laboratory at Shanghai Ocean University. The prawns were cultured in abundantly aerated and filtered freshwater and were fed using a commercial formula diet daily. Prawns were allowed to acclimate to their new environment for at least 2 weeks in culture conditions comprising 24.5 ± 0.5 °C, pH 7.8 ± 0.1, and DO 6.5 ± 0.2 mg/l. To analyze tissue distribution, hemolymph was extracted from 10 healthy prawns into a 1.5 ml Eppendorf tube and diluted using an equal volume of anticoagulant solution. Immediately, the hemocytes were subjected to centrifugation at 1000*g* for 10 min, and the supernatant was discarded. We also dissected out the hepatopancreas, gill, muscle, brain, and intestine from 10 prawns. Previously established techniques (65) were used for the primary culture of hemocytes. Primary hemocytes isolated from adult prawns were resuspended gently in Leibovitz’s L-15 medium (Sigma) containing 1% antibiotics (10,000 units/ml penicillin, 10,000 μg/ml streptomycin [Gibco]), and 0.2 mM NaCl, at pH 7.20 to 7.40. Insect S2 cells, as hemolymph-derived cells, were cultured in *Drosophila* Schneider’s medium (Gibco) supplemented with 10% fetal bovine serum and antibiotics. A hypoxic workstation, *In Vivo* 2500 (Ruskinn Technology), was used to expose the cultured cells to hypoxia.

### OCR determination

The cell numbers were as follows: 1 × 10^5^ for hemocytes and 1 × 10^5^ for *Drosophila* S2 cells. Glucose-containing XF base medium was used in the OCR analysis, following the manufacturer’s protocol (Seahorse Biosciences). In an XF Plasma Membrane Permeabilizer (Seahorse Biosciences), the sequential addition of antimycin A (4 mM) and ascorbate/tetramethyl-*p*-phenylenediamine (1 mM and 100 mM, respectively) allowed us to follow the electron flow through different complexes of the electron transport chain (66). Mitochondria were extracted from hemocytes, and ELISA kits (Nanjing Jiancheng Bioeng Institute) were used to assess the activities of mitochondrial respiratory chain complexes I, II, III, and IV, to further assess mitochondrial function.

### Hypoxia stress and sample collection

After acclimation, the control group were maintained in normoxic conditions (DO = 6.5 ± 0.2 mg/l). The addition of nitrogen gas allowed us to maintain a DO of 1.8 ± 0.1 mg/l over 24 h for the hypoxic group (48, 49). Next, 120 male prawns (3.28 ± 0.54 g) were assigned randomly and equally into six tanks (30 per tank, with the two treatments applied in triplicate): normoxia (control group) and hypoxia for 12 h. The hemocytes were sampled, frozen immediately, and placed at −80 °C for subsequent mRNA and protein extraction. All experiments were carried identically at least three times.

### RNA isolation, cDNA cloning, and bioinformatic analyses

A previously published protocol was used to extract RNA and synthesize cDNA for each sample (55). The *M. nipponense* mRNA encoding the *GLUT1* and *GLUT2* was identified in the *M. nipponense* transcriptome database SRP110812 at the National Center for Biotechnology Information. Procedures for cloning the *GLUT1* and *GLUT2* cDNA were performed as described previously (55). PCR products were purified and sequenced, and the obtained sequences were analyzed using ClustalW. SMART version 4.0 (https://www.ebi.ac.uk/Tools/msa/clustalw2/) was used to predict potential protein functional domains. The SWISS-MODEL server (https://swissmodel.expasy.org/) on ExPASy was used to predict the protein tertiary structure, which was visualized using the PyMOL software (https://pymol.org/2/). MEGA4 (www.megasoftware.net) was employed to construct the phylogenetic tree using the neighbor-joining method. Table S1 shows the corresponding accession numbers.

### Construction, transfection, and activity assays of luciferase reporter plasmids

The *M. nipponense GLUT1* and *GLUT2* promoters were serially truncated by PCR using primers P1, P2, P3, and P4 (Table S2). The truncated promoters were cloned into the firefly luciferase reporter vector pGL3-Basic *via* its KpnI/XhoI sites to produce plasmids pGL3-GLUT1-1, pGL3-GLUT1-2, pGL3-GLUT1-3, and pGL3-GLUT1-4. Similarly, we constructed plasmids pGL3-GLUT2-1, pGL3-GLUT2-2, and pGL3-GLUT2-3. The protocols detailed in a previous study were used for the luciferase assay and transfection experiments under normoxic and hypoxic conditions (56). Briefly, the pGL3-reporter plasmids containing one copy of each potential HRE site were transfected into hemocytes using the X-trem-eGENE HP DNA transfection reagent (Roche). Cells were then subjected to hypoxia treatment for 24 h. A Dual-Luciferase reporter assay system (Promega) was used to determine the firefly luciferase activity, which was normalized to the activity of Renilla luciferase as the internal control. A QuickChange Site-Directed Mutagenesis Kit (Stratagene) was used to mutate the *GLUT1* promoter HRE motifs using pairs of mutagenic primers, following the manufacturer's protocol. DNA sequencing was used to confirm the mutated HRE sites.

### Real-time qRT–PCR analysis

RNA was extracted from *M. nipponense* cells and tissues and converted to cDNA using reverse transcription. Real-time qPCR was then used to quantify the cDNA using the LightCycler system (Roche) and a SYBR Green kit (Takara). Table S2 lists the primers used to amplify the target genes and the mRNA encoding β-actin (as an internal control). Serial dilutions of pure cDNA samples were used to created standard curves from which each primer's amplification efficiency could be estimated (all amplification efficiency values ranged from 0.95 to 1.05). Target gene relative expression levels were determined using the 2^−ΔΔCT^ method (67).

### RNAi

Primers attached to the T7 promoter (Table S1) were used to amplify HIF-1α and *GLUT1* cDNA fragments, which were used as templates to produce siRNA employing an T7 transcription kit (Fermentas). A *GFP* siRNA from Gene Pharma Co was used as the control. *In vitro*, the siRNA (in RNase-free water) was transfected into primary-cultured *M. nipponense* hemocytes with the aid of 10 nM Lipofectamine 3000 (Thermo Fisher Scientific). *In vivo*, we injected the siRNA (2 μg/g) into prawn hemolymph. To lengthen the effect of RNAi, a second injection was delivered to each prawn at 48 h after the first injection. qRT–PCR was used to assess the efficiency of RNAi, with double-stranded GFP RNA as the control. After setting up the RNAi assay, hypoxia for 24 h was used to stimulate gene-silenced hemocytes *in vitro* or gene-silenced prawns *in vivo*.

### Prawn survival analysis

Prawns were as signed randomly to two groups (n = 20 per group). The prawns in the two groups were injected with the *GLUT1* siRNA or *GFP* siRNA, respectively. After siRNA-mediated *GLUT1* knockdown, all prawns were transferred into three hypoxia tanks. In each tank, dead prawns were counted daily for 5 days to assess survival.

### Flow cytometry analysis

After siRNA transfection, primary-cultured hemocytes were exposed to hypoxia for 24 h. An ROS assay kit including the oxidant-induced fluorescent probe 2′,7′-dichlorofluorescein-diacetate (Nanjing Jiancheng BIO, Inc) was used to determine the intracellular ROS concentrations in the hemocytes. Then, the transfected hemocytes were gently washed three times with PBS and assessed using flow cytometry (CytoFLEX S; Beckman Coulter) at gap 1, synthesis, and gap 2 phases. An Annexin V-enhanced GFP/propidium iodide apoptosis detection kit (KeyGEN Biotech) was used to assess apoptosis, and the apoptotic cells were detected using a flow cytometer (68). The experiments were carried out independently six times.

### Western blotting and immunofluorescence staining

Proteins were extracted from hemocytes following hypoxia treatment, and a Pierce bicinchoninic acid protein assay kit (Thermo Fisher Scientific) was used to determine the protein levels. Then, equal amounts of protein (50 μg) were separated using 10% SDS-PAGE, followed by electrophoretic transfer to a polyvinylidene fluoride membrane (Millipore) (69). Antibodies recognizing HIF-1α (ab51608), GLUT1 (ab115730), PDK1 (ab90444), and β-actin (ab8224) were purchased from Abcam. After confirming that these antibodies did not generate nonspecific signals, the membranes were incubated with the aforementioned antibodies (1:500 diluted). The membranes were washed three times using Tris-buffered saline, incubated with alkaline phosphatase–conjugated goat anti-rabbit immunoglobulin G (IgG) (1:10,000 diluted in Tris-buffered saline) for 3 h, and washed to remove unbound IgG. β-actin was used as the internal control. An enhanced chemiluminescence detection system was used to visualize the immunoreactive protein bands on the membranes, whose gray values were quantified using ImageJ software (version 1.51; National Institutes of Health), and values of GLUT1 were normalized to those of β-actin and expressed as a ratio of the normoxia control sample.

For immunofluorescence, the prawn hemocytes were fixed for 20 min in 4% paraformaldehyde in PBS. The fixed cells were subjected to transverse sectioning and mounted on slides. The slides were incubated with primary antibodies and then with labeled secondary antibodies. The cells were observed and imaged under an Olympus confocal laser scanning microscope (Olympus America, Inc) following the detailed protocol provided in our previous publication (68). For each group, six transverse sections of the hemocytes were quantified for GLUT1 and GLUT2 expression employing mean absorbance values. We subtracted the background values obtained from adjacent sections in the negative control group (incubated with normal rabbit IgG as the primary antibody) from each section. Values calculated from the replicates within the same group/condition were averaged to provide the mean absorbance levels.

### ChIP assay

ChIP was carried out using a ChIP assay kit (Millipore) following the manufacturer's protocol. ChIP was performed for hemocytes cultured under hypoxic or normoxic conditions using anti-HIF-1α antibodies. As a control for nonspecific genomic DNA binding, normal IgG was used. The DNA fragments pulled down using the anti-HIF-1α antibodies were subjected to conventional PCR (70).

### Glucose uptake, lactate production, and ATP generation assays

A 2-Deoxyglucose Glucose Uptake Assay Kit (ab136955; Abcam) kit was used to measure glucose uptake. In brief, cells were incubated with the glucose analog 2-deoxyglucose, and oxidation of the accumulated 2-DG6P generated NADPH, resulting in oxidation of a substrate. Hemocyte lactate levels were measured using a lactate content detection kit (Beijing Solarbio Science & Technology Co, Ltd) following the manufacturer’s protocol. The assay was carried out independently six times. The results were normalized according to each sample's protein concentration (Beyotime Institute of Biotechnology). Hemocyte ATP levels were assessed using an ATP content detection kit (Beijing Solarbio Science & Technology Co, Ltd) with respect to the supplier's guidelines. In brief, cells were lysed and subjected to centrifugation for 10 min at 10,000*g* and 4 °C. The supernatant was retained and incubated with ATP-detection buffer, which produced chromogenic products. The absorbance values of the chromogenic product were detected at 340 nm using a TECAN microplate reader (Tecan). The assay was carried out in triplicate, and the protein content of each sample was used to normalize the ATP level.

### Metabolic tracking test of ^14^C-labeled nutrients

After prawns were injected with 10 μg of siGLUT1 (with siGFP as a control), 36 prawns (12 prawns for each treatment group) were used to perform the metabolic tracking of ^14^C-labeled nutrient test, following a 24 h fast, with or without hypoxia treatment. The metabolic tracking test was determined by injecting [1-^14^C]-PA (PA^∗^) or D-[1-^14^C]-glucose (Glu∗) or L-[^14^C(U)]-amino acid mixture (AA^∗^) (PerkinElmer) on the base of the third pereiopod according to previous studies (71, 72). Afterward, these injected prawns were divided into three groups to perform three different radioactivity retention assays (four prawns for one assay, n = 4). The retention radioactivity was assayed as described previously (73).

### TUNEL assay

Prawn brain cell apoptosis was assessed using TUNEL assays for samples from prawns subjected to RNAi and hypoxia for 24 h. The cells were stained using the TUNEL reaction reagent, whereas their nuclei were counterstained using 4′,6-diamidino-2-phenylindole (R37606; Invitrogen). TUNEL-positive cells were counted under a fluorescence microscope (74). The assay was carried out independently six times.

### Statistical analysis

All data were analyzed statistically using SPSS software (version 19.0; IBM Corp), which are presented using the mean ± standard error. A two-tailed Student’s *t* test was used to compare data between two groups, whereas one-way ANOVA was used to compare data among more than two groups, as indicated in the figure captions.

## Data availability

All data are contained within the article.

## Supporting information

This article contains supporting information.

## Conflict of interest

The authors declare that they have no conflicts of interest with the contents of this article.
