# Supplementary material for: Glucose transporter GLUT1 expression is important for oriental river prawn (Macrobrachium nipponense) hemocyte adaptation to hypoxic conditions
Source: J Biol Chem. 2022 Nov 24;299(1):102748. doi: 10.1016/j.jbc.2022.102748 (PMC9758439; doi:10.1016/j.jbc.2022.102748)
Supplement: Supplemental Figures and Tables [file mmc1.doc]

Glucose transporter GLUT1 expression is important for oriental river prawn (*Macrobrachium nipponense*) hemocyte adaptation to hypoxic conditions

Xichao Sun^a,b^, Cheng Xue^a^, Yiting Jin^a^, Chao Bian^c^, Na Zhou^d^, Shengming Sun^a,b*^

^a^ Key Laboratory of Exploration and Utilization of Aquatic Genetic Resources, Ministry of Education, Shanghai Ocean University, Shanghai 201306, China

^b^ International Research Center for Marine Biosciences at Shanghai Ocean University, Ministry of Science and Technology, China

^c^ Shenzhen Key Lab of Marine Genomics, Guangdong Provincial Key Lab of Molecular Breeding in Marine Economic Animals, BGI Academy of Marine Sciences, BGI Marine, BGI, Shenzhen 518083, China.

^d^ State Key Laboratory of Quality Research in Chinese Medicine and School of Pharmacy, Macau University of Science and Technology, Macau, China

*Corresponding author: Shengming Sun

Key Laboratory of Exploration and Utilization of Aquatic Genetic Resources, Ministry of Education, Shanghai Ocean University, Shanghai 201306, China

E-mail: sunshengming621416@163.com. Tel.: +86 021 61908301

**Supplementary Tables**

**Table S1.** GLUT1/2 amino acid sequences from different species

| Gene | Species name | GenBank accession No |
| --- | --- | --- |
| GLUT1 | *Macrobrachium nipponense* | MT733824 |
|  | *Gadus morhua* | AAS17880.1 |
|  | *Cyprinus carpio* | AAF75683.1 |
|  | *Penaeus monodon* | AYF59257.1 |
|  | *Penaeus japonicus* | XP_042860524.1 |
|  | *Portunus trituberculatus* | MPC16930.1 |
|  | *Homo sapiens* | NP_006507.2 |
|  | *Mus musculus* | sp|P17809.4 |
|  | *Penaeus vannamei* | AIT97023.1 |
|  | *Equus caballus* | NP_001157443.1 |
|  | *Bubalus bubalis* | NP_001277799.1 |
|  | *Sus scrofa* | AMN88559.1 |
|  | *Oreochromis niloticus* | NP_001266656.1 |
|  | *Megalobrama amblycephala* | AKR15142.1 |
| GLUT2 | *Macrobrachium nipponense* | OM049769 |
|  | *Penaeus japonicus* | XP_042873725.1 |
|  | *Penaeus vannamei* | ROT70592.1 |
|  | *Portunus trituberculatus* | XP_045107890.1 |
|  | *Mus musculus* | NP_112474.2 |
|  | *Homo sapiens* | sp|P11168.1 |
|  | *Penaeus monodon* | XP_037805089.1 |
|  | *Sus scrofa* | AMN88560.1 |
|  | *Gadus morhua* | AAV63984.1 |
|  | *Trachinotus ovatus* | QWS64999.1 |
|  | *Danio rerio* | AAZ43092.1 |
|  | *Rattus norvegicus* | NP_037011.2 |
|  | *Oncorhynchus mykiss* | NP_001117761.1 |

**Table S2.** Primers used in this study

| Primer | Primer sequence (5′-3′) |
| --- | --- |
| **cDNA cloning** |  |
| GLUT1 (5′-RACE primer) | CGAAGATGGACACAGC |
| GLUT1 (5′-RACE primer) | ATGAGGTCTTGGATGCTG |
| GLUT1 (5′-RACE primer) | CTTCCAGCAGTCCCCTAT |
| GLUT1 (3′-RACE primer) | CGAGGAAATGCGAGCGGAGGAAGC |
| GLUT1 (3′-RACE primer) | ACCAGTCAGAATCGCACATGAGTA |
| GLUT2 (5′-RACE primer) | GCATTCAAACACGCTG |
| GLUT2 (5′-RACE primer) | CAGCGAAAAACTGCGTCC |
| GLUT2 (5′-RACE primer) | CTACCAGTTGCTCCGAGG |
| GLUT2 (3′-RACE primer) | TCTACCCAAATGTCAAGGAAGCCG |
| GLUT2 (3′-RACE primer) | CGCTCCCTTGAAGAATGCTATCCA |
| **Promoter Plasmid construction** |  |
| GLUT1-F1 (genome walking) | CTCCTTCCAGCAGTCCCCTA |
| GLUT1-R1 (genome walking) | GCGTTGATGACGCCCGTG |
| GLUT1-F2 (genome walking) | TGAATAAGACCAGGGCCTAACG |
| GLUT1-R2 (genome walking) | CGTCTGTCCATCATCTTC |
| P1-F (promoter activity)  −110 to 611 | GCTTTCCAACTCTTTTGGTGGAGGTAAAGAATAC |
| P1-R (promoter activity)  −110 to 611 | TACTAGCTTCTTTTCAGGAGCCTCGTTGACCATCTTCAT |
| P2-F (promoter activity)  −710 to 611 | GATTAAGAAATCAAAGTTAGCCTTTCTTTATCAGTTCGG |
| P2-R (promoter activity)  −710 to 611 | TACTAGCTTCTTTTCAGGAGCCTCGTTGACCATCTTCAT |
| P3-F (promoter activity)  −910 to 611 | CTTTACTTCGCGGATCGAATGAATTTTAATATATAT |
| P3-R (promoter activity)  −910 to 611 | TACTTCTACCAGTTGCTCCGAGGACTTTTCTTCGATCAT |
| P4-F (promoter activity)  −1460 to 611 | ATCGGTAATGTTTTGACTTCTGTCTCGTTCGAACATC |
| P4-R (promoter activity)  −1460 to611 | TACTTCTACCAGTTGCTCCGAGGACTTTTCTTCGATCAT |
| GLUT2-F1 (genome walking) | ATCGGTAATGTTTTGACTTCTG |
| GLUT2-R1 (genome walking) | CGGGCTTCTTCCATACTT |
| P1-F (promoter activity)  −320 to 352 | TTGTCTGTGATTAAATGTTCGCCGGGTGGGTGTCCC |
| P1-R (promoter activity)  −320 to 352 | AAGGTCAAACCCATGTTGTGCCCGCAGTAGTTGCG |
| P2-F (promoter activity)  −850 to 352 | TACACGATTAGAAGATCAACTCAGATAAAGTAGAT |
| P2-R (promoter activity)  −850 to 352 | AAGGTCAAACCCATGTTGTGCCCGCAGTAGTTGCG |
| P3-F (promoter activity)  −1513 to 352 | ACTTAACTATTAATATACCTTTGCCAGAA |
| P3-R (promoter activity)  −1513 to 352 | AAGGTCAAACCCATGTTGTGCCCGCAGTAGTTGCG |
| **RNA interference** |  |
| siRNA- HIF-1α-F | CUGAUGACCAGCAACUUGA |
| siRNA- HIF-1α-R | UCAAGUUGCUGGUCAUCAG |
| siRNA-GLUT1-F | GCAACUUUCGCAGCAGCUUTT |
| siRNA-GLUT1-R | AAGCUGCUGCGAAAGUUGCTT |
| siRNA-GFP-F | UAAUACGACUCACUAUAGGG |
| siRNA-GFP-R | CCCUAUAGUGAGUCGUAUUA |
| **Quantitative real-time PCR** |  |
| GLUT1-F (real-time primer) | CCAACGGGTGTCTGACACCTCC |
| GLUT1-R (real-time primer) | GCACCTACTGAAAATAGAGACA |
| HIF-1α-F (real-time primer) | ACTCGACAAGCAGACCTTCG |
| HIF-1α-R(real-time primer) | TGCATCTCGTAAAGGGACCG |
| GLUT2-F (real-time primer) | CATCAACCACATCGGCAGGAAGG |
| GLUT2-R (real-time primer) | CCCGTCGTTAGACCACACAAGATAC |
| PDK1-F (real-time primer) | ACAACAAGAGTAGCAGCAGGTCAAC |
| PDK1-R (real-time primer) | TTCATCCCGCTCCATTTCTTCATCC |
| HK-F (real-time primer) | GGGATGTTGTCGAGTTGCTC |
| HK-R (real-time primer) | TCGTCCAAATCACCATCCCA |
| LDH-F (real-time primer) | CTGTCCCAGTATGGTCAGGC |
| LDH-R (real-time primer) | CCGCATACACACTCCTCTGG |
| β-Actin F (real-time primer) | TGACCACTGCCGCCTCCTC |
| β-Actin R (real-time primer) | TGCCGCAAGATTCCATACCC |

**Supplementary Figures and legends**

**Figure S1**

**Figure S1. The deduced amino acid glucose transporter 1 (GLUT1) sequence in *Macrobrachium nipponense* is displayed above the nucleotide sequence.** The potential transmembrane motifs (TM) are shown in boxes. Black boxes show the conserved residues in all GLUT proteins, and the conserved residues of class I GLUTs are shown in gray boxes.

**Figure S2**

**Figure S2. The deduced amino acid glucose transporter 2 (GLUT2) sequence in *Macrobrachium nipponense* is displayed above the nucleotide sequence.** The potential transmembrane motifs (TM) are shown in boxes.

**Figure S3**

A


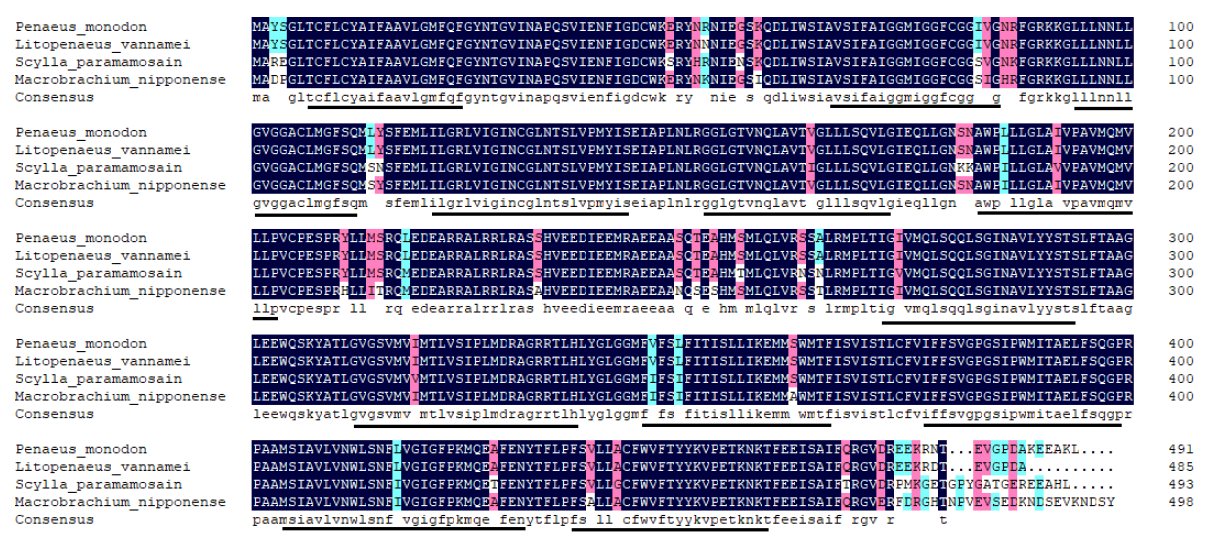


B


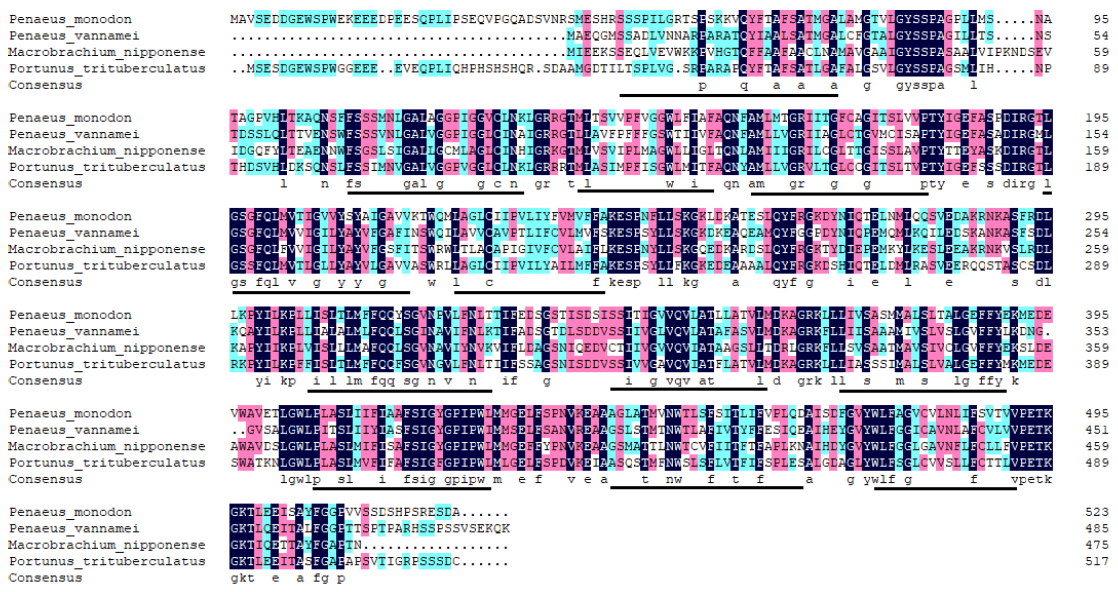


**Figure S3. Multiple sequence alignment analysis of the GLUT1 (A) and GLUT2 (B) from oriental river prawn with homologs in other crustacean.** Amino acid residues with 100% identity are shown in blue. The twelve conserved transmembrane motifs are underlined.

**Figure S4**


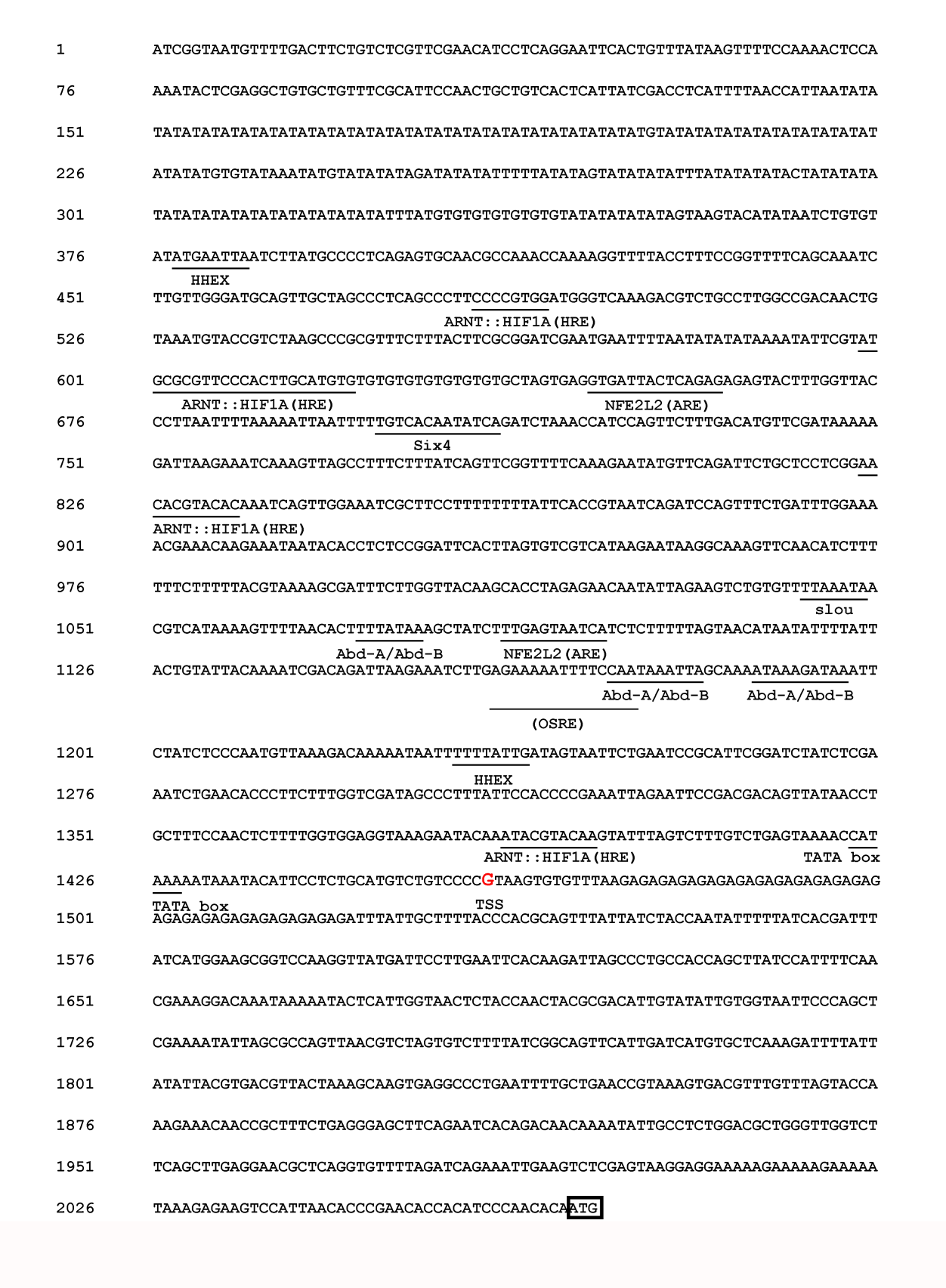


**Figure S4. The 5′-flanking sequence of the glucose transporter 1 (GLUT1) gene of oriental river prawn.** Putative transcription factor binding sites are underlined and labeled. Putative elements, including HIF-1a binding sites (HRE), are labeled. The stop codon (ATG) is boxed.

**Figure S5**


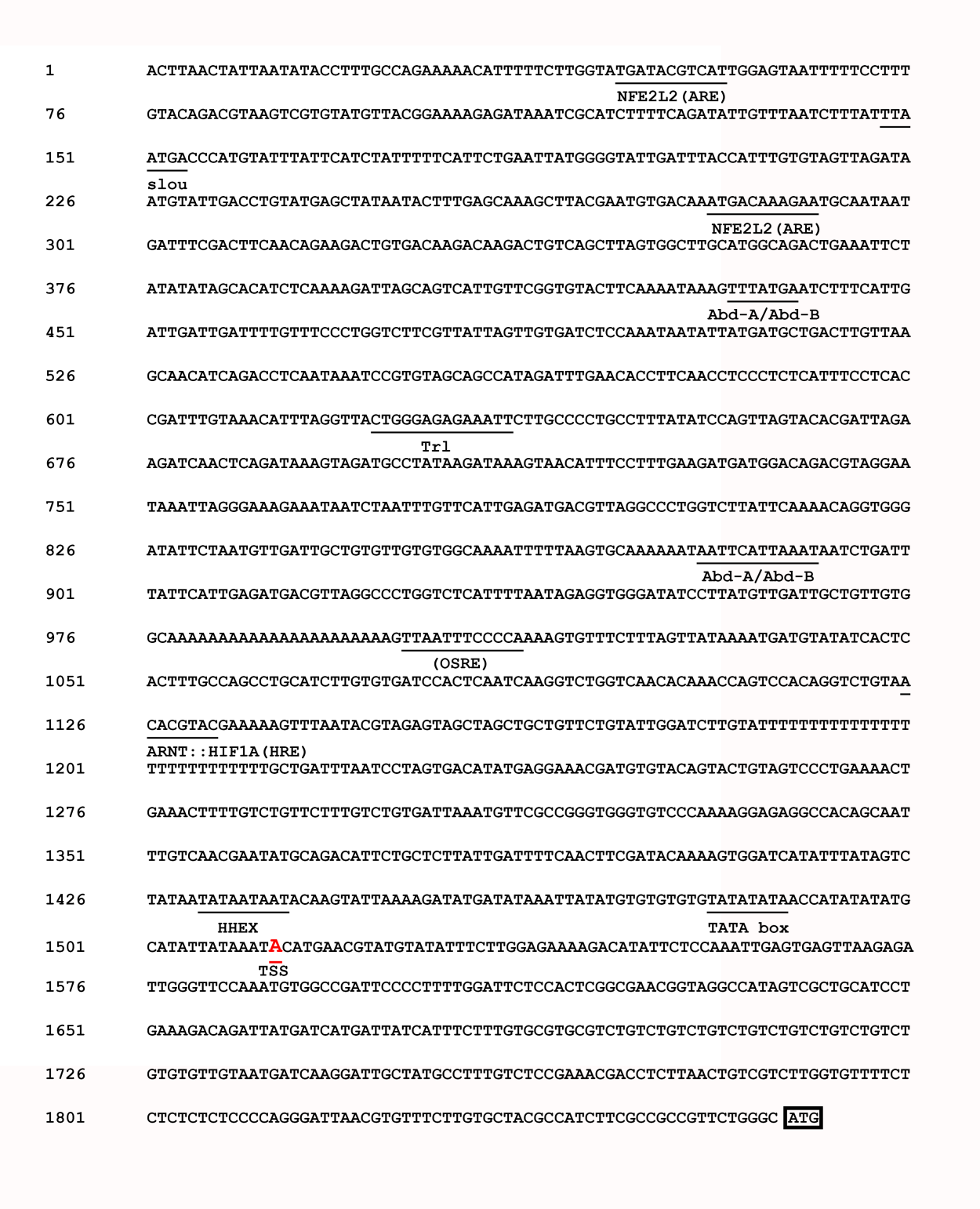


**Figure S5. The 5′-flanking sequence of the glucose transporter 2 (GLUT2) gene of oriental river prawn.** Putative transcription factor binding sites are underlined and labeled. Putative elements, including HIF-1a binding sites (HRE), are labeled. The stop codon (ATG) is boxed.

**Figure S6**

**A**


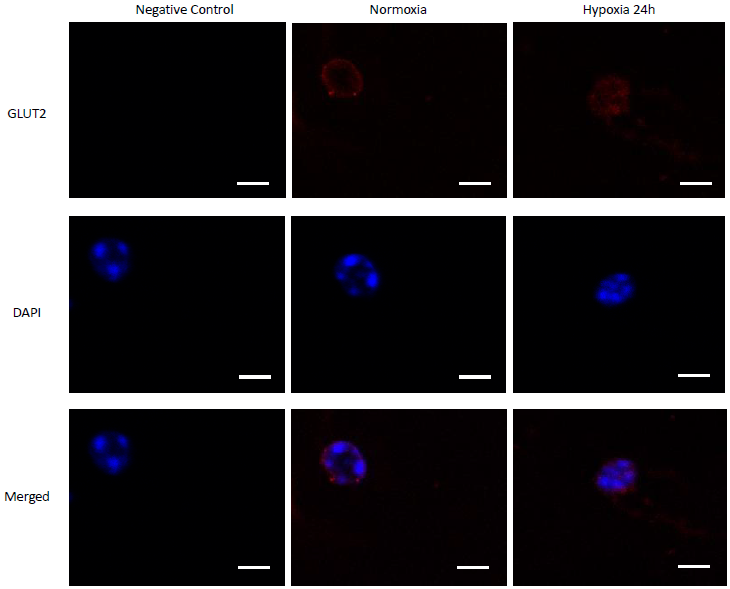


**B**


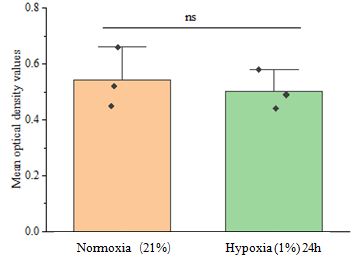


**Figure S6. Immunofluorescence of GLUT2 in the hemocytes of the oriental river prawn under hypoxia for 12 h.** *A*, Fluorescence was developed using secondary antibodies conjugated with Alexa 568 (red). Nuclei were stained with 4', 6-diamidino-2-phenylindole (DAPI) (blue). A negative control represents that the primary antibodies were replaced with normal non-immune serum. The white line in the lower right corner represents the scale bar (50 μm). *B*, Mean optical density values used for quantification of the expression of GLUT2 in response to hypoxia for 12 h. All values are presented as the mean ± S.E (N = 3); **p* < 0.05.

**Figure S7**


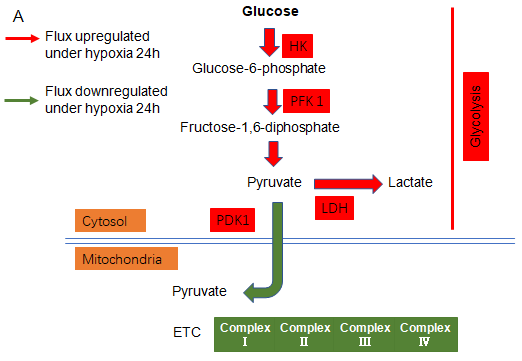


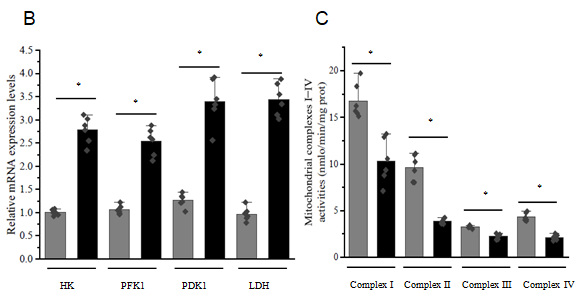


**Figure S7. The gene expression levels of glycolytic enzymes and mitochondrial complexes I−IV activities in prawn hemocytes under hypoxia.** *A*, A schematic of the metabolic pathway. Glucose enters prawn hemocytes through glucose transporters (GLUT1) and is converted to glucose-6-phosphate, fructose-6-phosphate, and fructose-1, 6-diphosphate by glycolytic enzymes. *B*, Shown are the gene expression levels of glycolytic enzymes. *C*, Shown is the measured mitochondrial complex I−IV activities. Results are the combined data of three experiments with different cell preparations and each value represents mean ± S.E (N = 3); **p* < 0.05. Black columns represent the hypoxia group, gray columns represent the normoxia control group.

**Figure S8**

Chamber O_2_


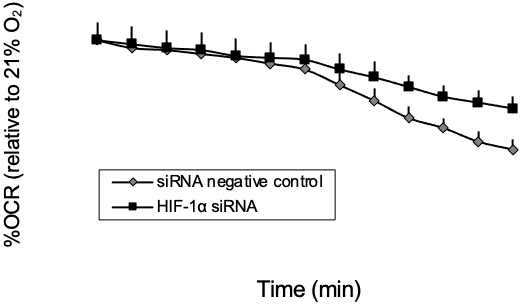


21%

11%

8%

4%

1%

*

*

*

*

**Figure S8. Hypoxia-induced glycolytic reprogramming was observed in *Drosophila* S2 cells (a kind of hemolymph-derived cell).** The proportion of oxygen consumption rate (%OCR) of hepatocytes in mild hypoxia was plotted as % relative to 21% O_2_ (y-axis) vs. time (x-axis). Results are combined data of three experiments with different cell preparations and each value represents mean ± S.E (N = 3); **p* < 0.05 Control RNAi versus HIF-1α RNAi; 21%, 11%, 8%, 5%, 1%, represent O_2_ concentrations.
